# CRISPR/Cas9-mediated gene modification and gene knock out in the human-infective parasite *Trichomonas vaginalis*

**DOI:** 10.1038/s41598-017-18442-3

**Published:** 2018-01-10

**Authors:** Brian D. Janssen, Yi-Pei Chen, Brenda M. Molgora, Shuqi E. Wang, Augusto Simoes-Barbosa, Patricia J. Johnson

**Affiliations:** 1 Department of Microbiology, Immunology & Molecular Genetics, University of California, Los Angeles, California, USA; 2 Molecular Biology Institute, University of California, Los Angeles, California, USA; 3 School of Biological Sciences, University of Auckland, Auckland, New Zealand

## Abstract

The sexually-transmitted parasite *Trichomonas vaginalis* infects ~1/4 billion people worldwide. Despite its prevalence and myriad adverse outcomes of infection, the mechanisms underlying *T. vaginalis* pathogenesis are poorly understood. Genetic manipulation of this single-celled eukaryote has been hindered by challenges presented by its complex, repetitive genome and inefficient methods for introducing DNA (i.e. transfection) into the parasite. Here, we have developed methods to increase transfection efficiency using nucleofection, with the goal of efficiently introducing multiple DNA elements into a single *T. vaginalis* cell. We then created DNA constructs required to express several components essential to drive CRISPR/Cas9-mediated DNA modification: guide RNA (gRNA), the Cas9 endonuclease, short oligonucleotides and large, linearized DNA templates. Using these technical advances, we have established CRISPR/Cas9-mediated repair of mutations in genes contained on circular DNA plasmids harbored by the parasite. We also engineered CRISPR/Cas9 directed homologous recombination to delete (i.e. knock out) two non-essential genes within the *T. vaginalis* genome. This first report of the use of the CRISPR/Cas9 system in *T. vaginalis* greatly expands the ability to manipulate the genome of this pathogen and sets the stage for testing of the role of specific genes in many biological processes.

## Introduction

*Trichomonas vaginalis* is an obligate extracellular, unicellular flagellated protozoan parasite and the causative agent of trichomoniasis, an infection that afflicts ~¼ billion people worldwide^1^. Infection can be asymptomatic or result in a variety of negative outcomes in women and men including vaginitis, cervicitis, pelvic inflammatory disease, prostatitis and urethritis^2,3^. Alarmingly, public health studies have reported an increased incidence of drug resistance to the drug used to treat infections, metronidazole^4–7^. Despite being a very common sexually transmitted infection, the basic mechanisms underlying pathogenesis and drug resistance are poorly understood. This is in part due to the slow adoption of molecular tools to study the genes and proteins involved in these processes^8,9^. The development and application of cutting-edge gene technologies to manipulate this medically-important parasite are needed to further advance our knowledge of disease mechanisms.

Studies aimed to test the function of *T. vaginalis* genes have been limited by inefficient methods of genomic manipulation. Most reports have relied on isolates naturally lacking expression of particular genes^10–22^ or those directed by lab-acquired mutations^23–27^ that result in changes in gene expression. Only two studies have reported gene knockout of non-essential proteins by integrating a drug resistance selection cassette in the gene locus relying purely on homologous recombination^28,29^. A few reports have also utilized knockdown-based strategies utilizing antisense RNA expression^30,31^ and modified oligonucleotides^32,33^. Knockdown techniques do not result in elimination of gene expression, which is often required to effectively test gene function, limiting the usefulness of this technology.

Recent advances in *in* vivo gene modification techniques have increased the ability to test gene function in both model and non-model organisms. The CRISPR (clustered regularly interspaced short palindromic repeats) system has recently revolutionized the ability to specifically target genes for modification in many organisms^34–37^. The CRISPR system is composed of two components, the Cas9 double stranded DNA nuclease of *Streptococcus pyogenes* and a user-customized version of its cofactor (guide RNA) to selectively target genes and induce double stranded DNA breaks. The double stranded breaks are repaired by non-homologous end joining (NHEJ), microhomology-mediated end joining (MMEJ) or homology-directed repair (HDR) pathways^36,38^. In many organisms, Cas9-gRNA expression induces repair of the double stranded break by the NHEJ and MMEJ pathways, which can result in insertion (NHEJ) or deletions (NHEJ and MMEJ) of nucleotides that may cause gene disruption and loss-of-function mutations. A more directed approach utilizes Cas9-gRNA in combination with a provided DNA template containing homology to the repair site to direct specific user-defined gene modifications. This approach requires a small region of donor DNA homology around the site of modification. More recently, CRISPR-based techniques have been applied to modify genes in a variety of parasitic protozoa^39–50^ and have been proposed for multicellular parasites^51^.

Here we report the first application of CRISPR/Cas9 to alter genes in *T. vaginalis*. We demonstrate Cas9-gRNA directed modification of a nanoluciferase reporter gene using homology directed repair. Cas9-gRNA mediated knockout of two endogenous *T. vaginalis* genes by replacement with a drug resistance gene cassette were also achieved. This report lays the foundation for further development and utilization of this CRISPR/Cas9 to rigorously test gene function in this understudied parasite.

## Results

### Expression of Cas9 and gRNA in *Trichomonas vaginalis*

**Figure 1:**
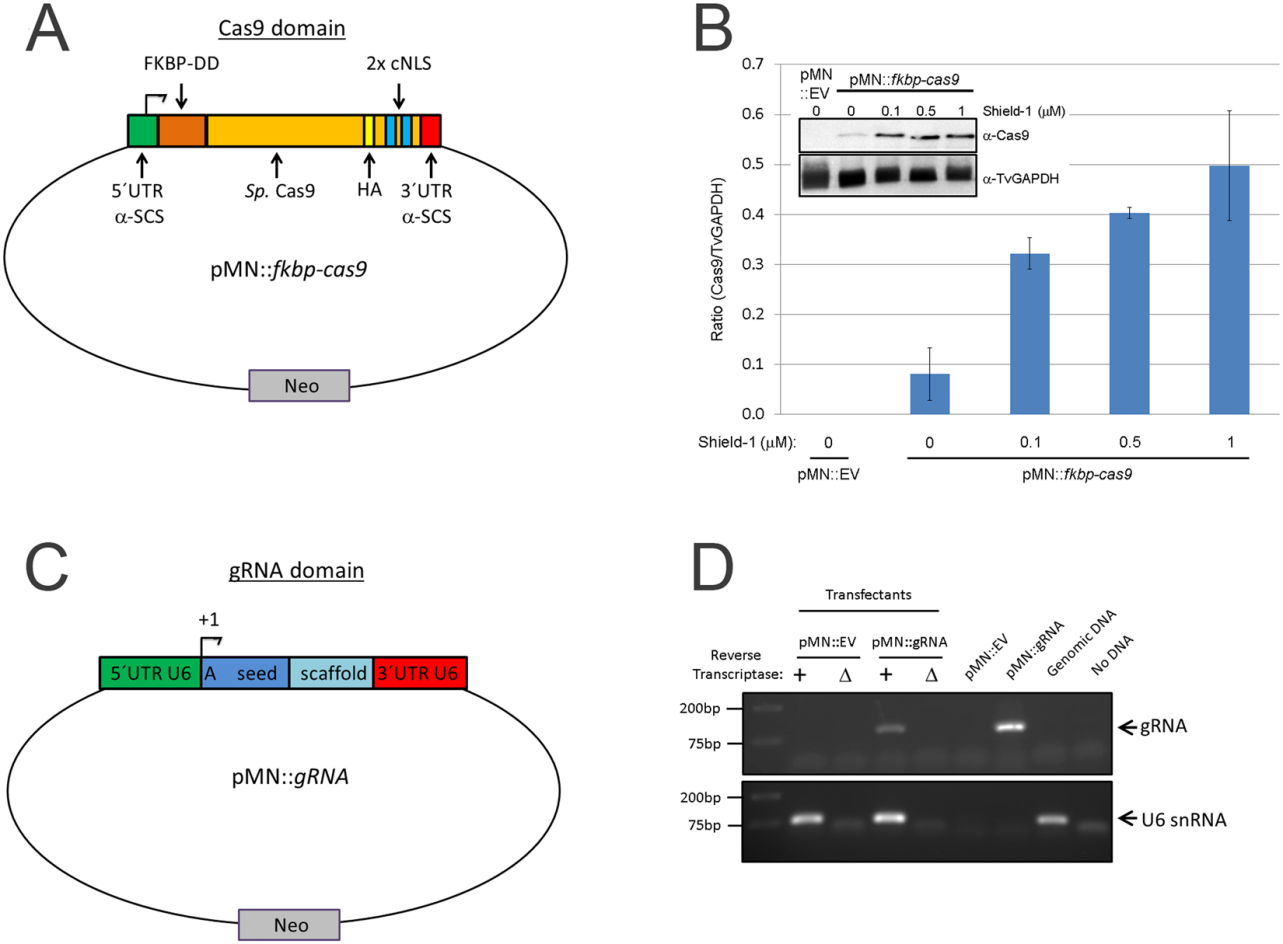
Setup of pMN::*fkbp-cas9* and pMN::gRNA constructs. (**A**) Cartoon representation (not to scale) of pMN::*fkbp-cas9* plasmid. The *T. vaginalis* gene α-SCS 5′ and 3′ UTRs of the pMasterNEO plasmid (pMN)^53^ drive expression of the *fkbp*(FKBP destabilization domain)-*cas9* fusion gene. The *cas9* variant used is the human codon optimized version with 2 SV40 nuclear localization signals at the C-terminus^56^. (**B**) Representative immunoblot analysis (inset) and quantification of replicate samples of pMN::*fkbp-cas9* expression. Samples were induced for 24 hours with 0, 0.1, 0.5 or 1 μM Shield-1 ligand before protein was collected and resolved by SDS-PAGE. Inset, upper: anti-Cas9 immunoblot (Clontech); Inset, lower: anti-TvGAPDH antibody (Cocalico Biologicals). Control sample used a pMN::EV (pMasterNEO::empty vector) transfectant. Quantitative comparison of samples utilized normalized signal of Cas9 (upper blot) to GAPDH signal (lower blot) taken from within an experiment and analyzed in parallel on separate immunoblots using a Bio-Rad Gel Doc and ImageLab software. Bar graph represents average +/− standard deviation of two independent analyses. (**C**) Cartoon representation (not to scale) of pMN::*gRNA* – 360 bp of the *T. vaginalis* U6 5′ UTR and 37 bp of the 3′ UTR flank the gRNA. A 20 nt seed region with the first nucleotide always an adenine residue followed by 19 nt seed region used for specific targeting and the gRNA scaffold. (**D**) RT-PCR products amplifying either the gRNA sequence or the U6 snRNA (control). Total RNA was subjected to +/− reverse transcriptase then amplified by PCR using gRNA- or U6 snRNA-specific primers. Control PCRs included the pMN::EV and pMN::*gRNA* plasmids, genomic DNA and no DNA template. PCR products were imaged using a Bio-Rad Gel Doc and ImageLab software. Full length blots/gels are presented in Supplementary Figure S5.

To date only two genes are reported^28,29^ to have been deleted (i.e. knocked out) from the haploid genome^52^ of the human parasite *T. vaginalis*. In order to improve the ability to modify *T. vaginalis* genes, we have developed the use of the CRISPR/Cas9 system as a means to directly modify genes in this parasite. One technical problem encountered when developing CRISPR/Cas9 in several systems is the toxicity associated with Cas9 expression *in vivo* ^43^. In initial experiments in which *cas9* was constitutively expressed using our standard *T. vaginalis* expression vector (pMasterNEO)^53^ and selection with G418, we were unable to select viable parasites, consistent with possible Cas9 toxicity. To address whether Cas9 was toxic in *T. vaginalis* cells selected for stable expression, we cloned the *cas9* gene as a fusion protein with an N-terminal FKBP destabilization domain (FKBP-DD). The presence of this domain on a protein results in the degradation of the protein in the absence of the stabilizing ligand, Shield-1^54,55^. We also added both a hemagglutinin (HA) tag and two SV40 C-terminal nuclear localization motifs^56^ at the 3′ end of Cas9, to allow detection of the protein by immunoblot and to direct it into the nucleus, respectively (pMN:*fkbp-cas9*; Fig. 1A). Using this construct, we were able to transfect and G418 select for parasites with the pMN::kfbp-Cas9 plasmid in the presence or absence of the stabilization ligand Shield-1. Regulating the expression of Cas9 using the FKBP-DD and Shield-1 greatly reduced, but did not eliminate, basal expression of the FKBP-Cas9 protein in the absence of Shield-1 (Fig. 1B). We found that a low concentration of Shield-1 (0.1 μM) resulted in ~4-fold increased stabilization of the FKBP-Cas9 protein compared to cultures lacking Shield-1. A greater than 6.5-fold increase was observed using 1 μM Shield-1 (Fig. 1B). Moreover, we noticed no growth difference in parasites containing the pMN::*fkbp-cas9* plasmid compared to parasites transfected with an empty vector (pMN::EV) when grown in the absence of Shield-1 (data not shown). Upon addition of Shield-1 parasite numbers were reduced in the pMN::*fkbp-cas9* transfectants compared to the pMN::EV parasites which did not exhibit growth rate reduction (data not shown), confirming the initial suspicion that Cas9 might exhibit toxicity in *T. vaginalis*.

We then made a construct that would allow the expression of gRNA(s) using the pMasterNEO plasmid backbone (Fig. 1C; see Methods for details). The gRNA construct (pMN::gRNA) contains the 360 bp sequence upstream of the U6 transcription start site (TSS), and is flanked at the 3′ end by the original 37 bp downstream of the U6 transcription unit to ensure proper transcription termination^57^. Between these 5′ and 3′ regulatory elements, we inserted the first 20 nt sequence of the U6 snRNA transcript (5′- AGCTGAGGATATGGCCTAGT)^57^ followed by the conserved 76 bp scaffold sequence necessary for Cas9 targeting specificity and gRNA interaction, respectively (Fig. 1C). To evaluate whether transcription of this small RNA (a gRNA mimic) would be driven by the putative *T. vaginalis* U6 promoter, we performed RT-PCR on total RNA extracts from cells transfected with this construct (pMN::gRNA). Transcription was only detected from cells transfected with this construct (Fig. 1D) and was absent in negative control samples, transfected with an empty vector (pMN::EV). Next, sequential mutagenesis was done to replace the first 20 nt of *T. vaginalis* U6 snRNA, except for the first adenosine. None of the nt substitutions had an effect on transcription by end-point RT-PCR (data not shown). While the first transcribed nucleotide in the U6 transcript of metazoans and plants is typically a guanosine, *T. vaginalis* uses an adenosine^57^. Since the first transcribed nucleotide is necessary for U6 transcription in other systems^58^, but dispensable for the DNA target specificity of the Cas9 gRNA^59^, we did not alter the +1 adenosine in our gRNA construct. The ability of the 360 nts upstream of the *T. vaginalis* U6 gene to drive transcription of an internally altered U6 gene indicates that the *T. vaginalis* U6 gene uses an upstream promoter as is found in metazoans and plants, and does not rely on internal promoter sequences as described for some unicellular eukaryotes (e.g. yeast and trypanosomes)^60–62^. These data also demonstrate that the *T. vaginalis* U6 promoter can be used to drive transcription of a sequence-customizable gRNA. In metazoan and plants, the core promoter of the U6 snRNA gene sits within the first ~70 bp upstream of the TSS^60^. In *T. vaginalis*, the core promoter of the U6 snRNA gene is unknown however we observed by end-point RT-PCR that there was no effect on transcription when the upstream region was reduced from 360 bp to 131 bp (data not shown). All additional gRNA plasmids produced contain the 360 nt sequence 5′ of the U6 gene (Fig. 1C) preserving the +1 adenosine followed immediately by a 19 nt seed region that is customized to target the gene of interest.

### Increasing transfection efficiency of *Trichomonas vaginalis*

**Figure 2:**
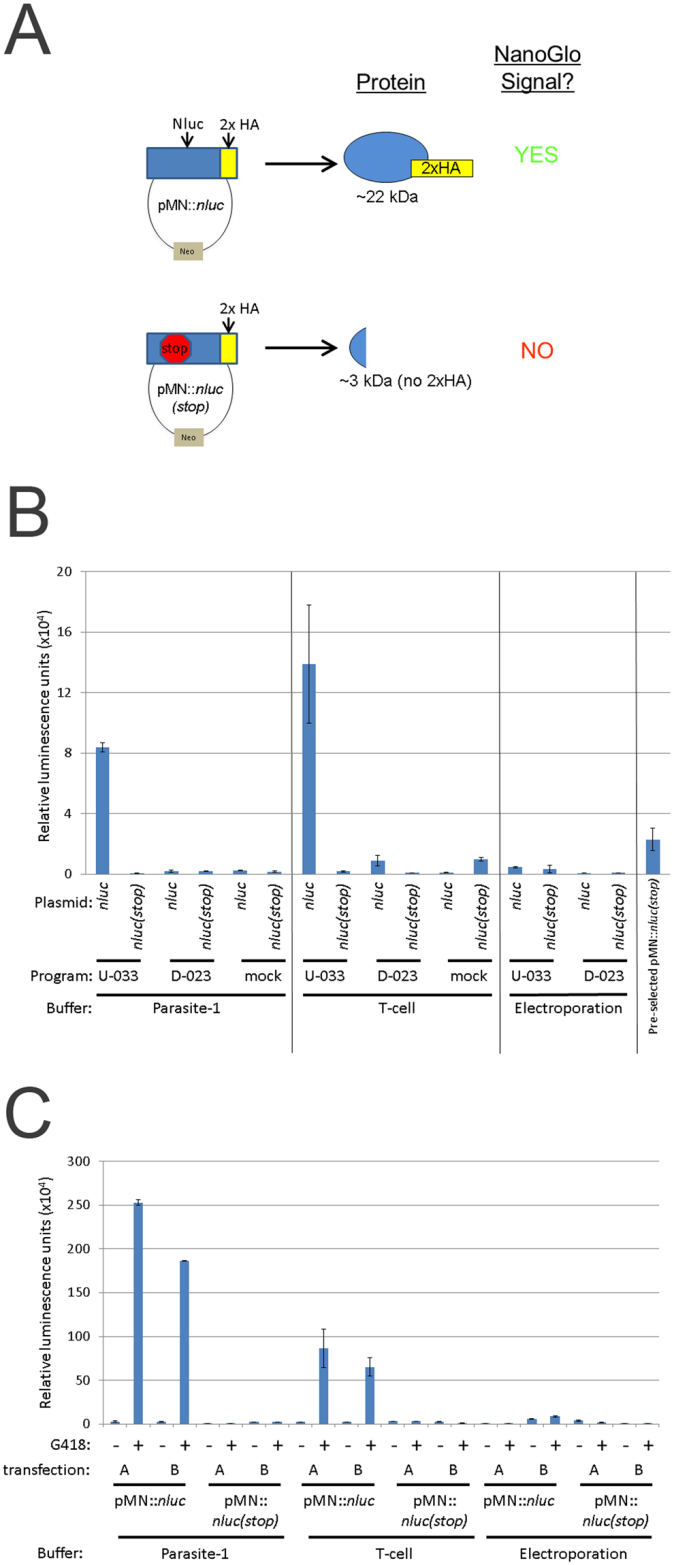
Parameters for transfection of *Trichomonas vaginalis* for detection of nanoluciferase activity. (**A**) Cartoon representation of nanoluciferase plasmids and predicted outcome for protein production. (**B**) A total of 1 × 10^7^ parasites were either nucleofected using the U-033 (“U”) or D-023 (“D”) programs (Amaxa) or mock (no nucleofection) with 10 μg of pMN::*nluc* or pMN::*nluc(stop)* in buffers Parasite-1 or T-cell (Lonza) or electroporated (975 μF, 350 V, Bio-Rad) in completed TYM media. Plasmids are pMN::*nluc*, denoted as “*nluc*” and pMN::*nluc(stop)*, denoted as “*nluc(stop)*” where indicated. Parasites were immediately recovered in completed TYM media. After 24 hours, a total of 1 × 10^6^ parasites were collected and nanoluciferase activity was measured by NanoGlo assay (Promega). Graph represents the average relative luminescence value +/− standard deviation of two transfections per condition. (**C**) Samples transfected with the pMN::*nluc* or pMN::*nluc(stop)* constructs and the U-033 program from Fig. 2B were subjected to G418-selection and allowed to grow for an additional +24 hours (+48 hours total, post-transfection). Equivalent numbers of parasites (1 × 10^6^) were assayed for nanoluciferase activity and the graph represents the average relative luminescence value +/− standard deviation of two attempts per condition.

Previous reports on transfection of *T. vaginalis* have used electroporation to deliver plasmids, linearized DNA or small oligonucleotides into the cell^53^. Although sufficient to introduce DNA, transfection efficiency using this method is low. With the need to introduce multiple DNAs into the same cell to achieve CRISPR/Cas9 editing, we sought to improve the transfection efficiency using the Amaxa nucleofection system^39,63–65^. We first compared electroporation versus nucleofection using the nanoluciferase reporter system^47,63^. Nanoluciferase (also termed NanoLuc) is a newly developed luciferase gene from a deep sea shrimp that produces stronger bioluminescence than the firefly luciferase (Promega). After normalization of parasite numbers, we subjected cells to either electroporation using our previously described protocol^53^ or nucleofection to introduce the wild-type nanoluciferase gene with a duplicate hemagglutinin (HA) tag at the 3′end (pMN::*nluc*) (Fig. 2A). To optimize nucleofection, multiple programs and buffers were tested. Two of the programs tested (D-023 and X-001) and buffer (Parasite-2) resulted in detection of nanoluciferase signal compared to controls, however the signal was less than that obtained using standard electroporation (Supplemental Figure 1 and Fig. 2B). We found that one program (U-033) yielded better nanoluciferase signal, although parasite survival was low (<25% of untransfected controls at +24 hours post transfection, comparable to electroporation). Increased nanoluciferase signal was observed for two nucleofection buffers tested, T-cell and Parasite-1. As predicted, cells transfected with the pMN::*nluc(stop)* plasmid containing a premature stop codon that eliminates nanoluciferase protein production (Fig. 2A and B) produced only background signals. In comparison to the signal intensity achieved using nucleofection we found that nanoluciferase signals measured from parasites electroporated with pMN::*nluc* barely increased over background signal (Fig. 2B). This is consistent with the low frequency of transfection of parasites by electroporation, 3% at best for surviving cells^66^. Nucleofection with the U-033 program increased nanoluciferase signal 18-fold using Parasite-1 buffer and 30-fold using T-cell buffer, relative to signals detected using electroporation.

To determine if signal was stable and detectable at later time points, we passaged the parasites in the presence or absence of 100 μg/ml G418 to select for the pMN::*nluc* and pMN::*nluc(stop)* plasmids (Fig. 2C). We noted that G418 selection for the pMN::*nluc* plasmid resulted in high levels of detectable nanoluciferase activity compared to transient nucleofection (Figures S2 and S3). The overall nanoluciferase signal for the unselected pMN::*nluc* parasites was reduced at +48 hours post-nucleofection compared to +24 hours. Selection for parasites harboring the pMN::*nluc* plasmids using G418 resulted in a dramatic increase with greater than 20 fold higher nanoluciferase signal compared to those transiently nucleofected (Fig. 2C). Electroporation did not yield the same increase (average ~1.2-fold) over the unselected counterparts (Fig. 2C and Fig. S3). Based on these data, we selected the U-033 program and the T-cell buffer as the optimal conditions to use for nucleofection of this strain of *T. vaginalis*. Together, these data demonstrate that nucleofection greatly improves transfection efficiency of *T. vaginalis*.

### CRISPR repair of a dead-luciferase gene in *Trichomonas vaginalis*

**Figure 3:**
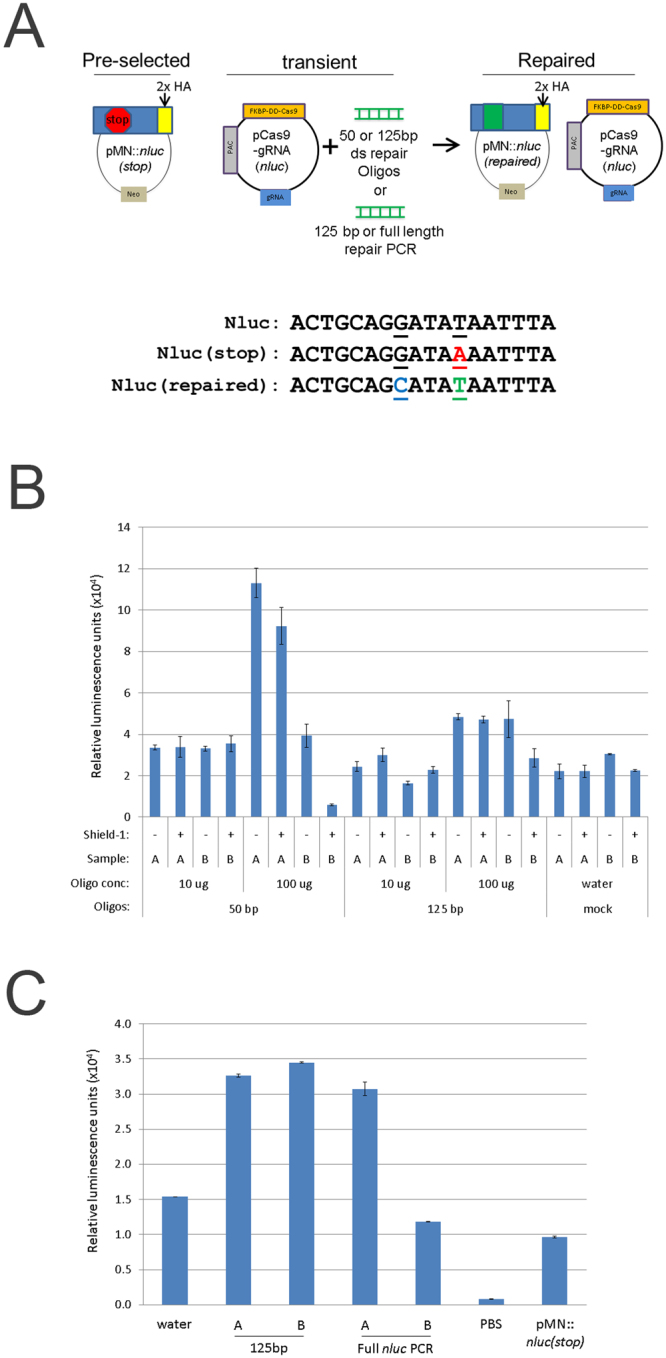
Nanoluciferase repair assay. (**A**) Upper: cartoon representation of nanoluciferase repair assay. Below: Cas9-gRNA(*nluc*) editing sites of *nluc(stop)* with mutation to create a premature stop codon (red, underlined). Also represented are the wild-type (*nluc*) and repaired (*nluc(repaired)*) versions. (**B**) A total of 5 × 10^7^ parasites pre-selected for the pMN::*nluc(stop)* plasmid were nucleofected in T-cell buffer using the U-033 program (Amaxa) with 10 μg or 100 μg of annealed repair oligos and 10 μg of pCas9-gRNA(*nluc*). Oligos contained the repair sequence and either 50 bp or 125 bp annealed repair oligos. Parasites were immediately recovered in completed TYM media +/− 1 μM Shield-1 and assayed for nanoluciferase activity after 24 hours. Equivalent numbers of parasites (1 × 10^6^) were assayed for nanoluciferase activity and the graph represents the average relative luminescence value +/− standard deviation for each condition. (**C**) A total of 5 × 10^7^ parasites pre-selected for pMN::*nluc(stop)* were nucleofected in T-cell buffer using the U-033 program (Amaxa) with 10 μg pCas9-gRNA(*nluc*) and either 100 μg of PCR-amplified 125 bp repair sequence (equivalent to ds repair oligos) or the full length *nluc* (“Full *nluc* PCR”) sequence. Parasites were immediately recovered in  completed TYM media + 1 μM Shield-1 and assayed for nanoluciferase activity after 24 hours. Equivalent numbers of parasites (1 × 10^6^) were assayed for nanoluciferase activity and the graph represents the average relative luminescence value +/− standard deviation for each condition.

To test the activity of Cas9, we used the highly-sensitive nanoluciferase repair assay originally described by Striepen and colleagues^47^. To express the DD- Cas9 and gRNAs together in *T. vaginalis*, we first constructed a plasmid containing both elements as well as a puromycin acetyltransferase (PAC) gene, pCas9-gRNA(*nluc*) (Fig. 3A). As outlined in Fig. 2A, we then preselected parasites containing the plasmid that expresses a dead luciferase (i.e. pMN::*nluc(stop)*) using G418. We subsequently co-nucleofected preselected parasites with the pCas9-gRNA(*nluc*) plasmid expressing a gRNA targeting the premature stop codon (Y18ochre) region of the gene and pre-annealed oligos capable of both repairing the stop codon (“repaired”, ochre18Y) and introducing a mutation that eliminates the PAM (AGG- > AGC). Mutation of the PAM requires the correction of two sites by oligo template-directed repair, increasing the confidence that repair is Cas9-gRNA directed, and not due to random point mutation that changed a stop codon back to a tyrosine codon. Mutation also prevents further targeting of the repair site by Cas9-gRNA(*nluc*). Transient nucleofection was used, without drug selection for expression of the gRNA or Cas9. Using this approach, we were able to demonstrate that CRISPR/Cas9 is capable of repairing the mutation to allow detection of nanoluciferase signal above background levels in *T. vaginalis* (Fig. 3).

The nanoluciferase repair assays were done using different lengths of oligos (50 nt and 125 nt) and we found that although the 50 bp annealed oligos yielded similar or better efficiency of repair, more variability was also observed (Fig. 3B). We also tested the use of PCR products corresponding to the 125 bp annealed oligos and the full length *nluc* gene. These also repaired pMN::*nluc(stop)*, restoring nanoluciferase activity (Fig. 3C) however, the efficiency was not increased.

**Figure 4:**
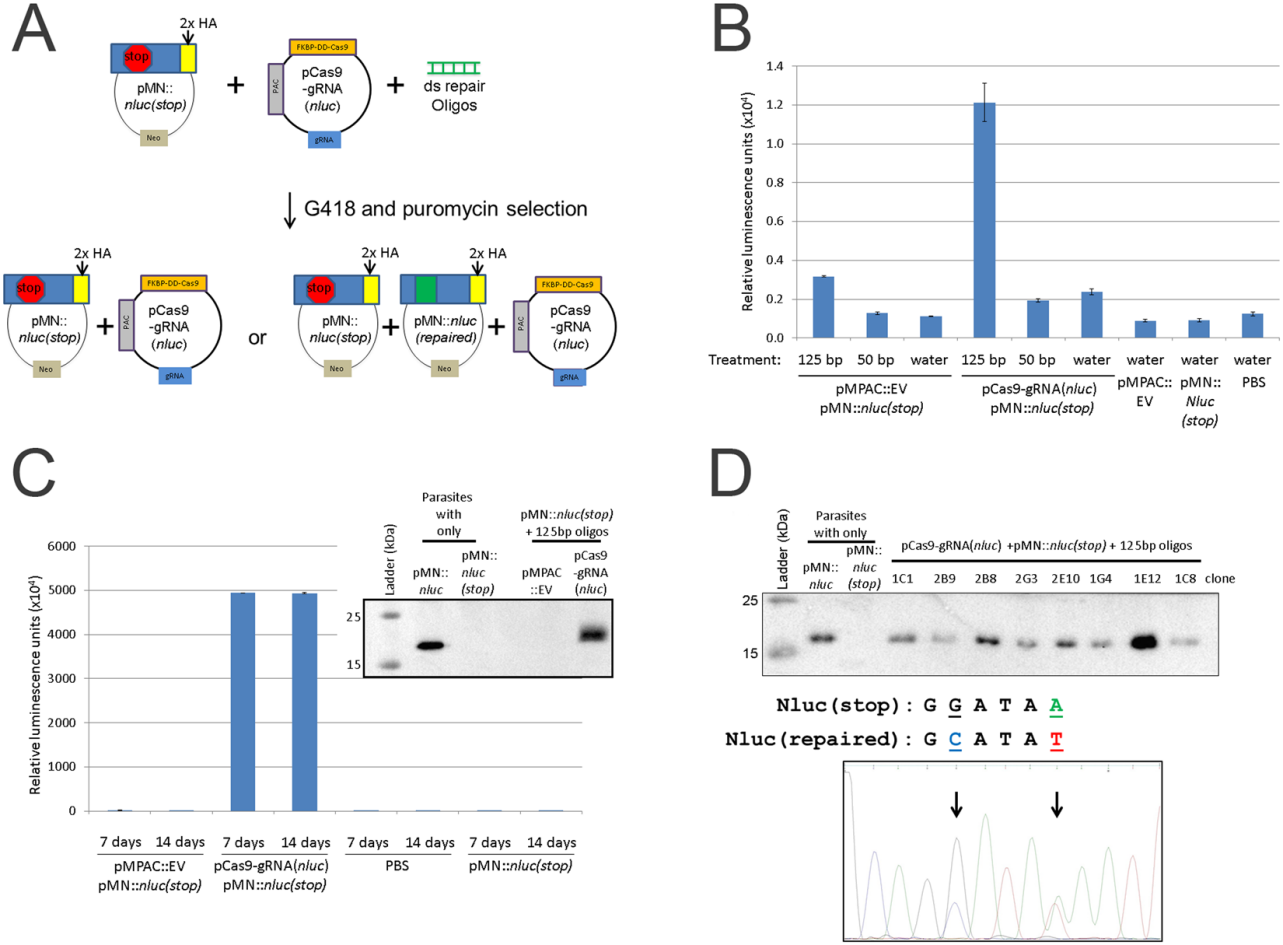
Transient transfection, selection and screening for nanoluciferase repair. (**A**) Cartoon representation of transient nanoluciferase repair activity assay followed by selection with puromycin and G418. (**B**) A total of 5 × 10^7^ parasites were nucleofected with 10 μg of pCas9-gRNA(*nluc*), 10 μg pMN::*nluc(stop)* and 100 μg of either 50 bp or 125 bp annealed repair oligos or water using T-cell buffer (Lonza) and the U-033 program (Amaxa). Parasites were immediately recovered in completed TYM media + 1 μM Shield-1. After 24 hours of recovery, equivalent numbers of parasites (1 × 10^6^) were assayed for nanoluciferase activity and the graph represents the average relative luminescence value +/− standard deviation from duplicate samples for each condition (**C**). After 24 hours of recovery, one transfection population of the pMPAC::EV- and pCas9-gRNA(*nluc*)-treated parasites from Fig. 4B was treated with 30 μg/ml puromycin and 50 μg/ml G418 to select for parasites transfected with both plasmids. After 7 and 14 days (post-nucleofection), parasites were re-tested for nanoluciferase activity by assay of equivalent numbers of parasites (1 × 10^6^). The graph represents the average relative luminescence value +/− standard deviation for each condition. Inset: Anti-HA epitope immunoblot analysis of control samples (pre-selected parasites) compared to protein isolated from puromycin/G418 selected parasites grown for two weeks. (**D**) Upper: anti-HA epitope immunoblot analysis of controls (pre-selected plasmids) and representative clones from 14 day puromycin/G418 selected pCas9-gRNA(*nluc*) + pMN::*nluc(stop)* + 125 bp oligo parasites in Fig. 4C. Lower: representative DNA sequencing trace (Genewiz sequencing and FinchTV sequence viewer) of PCR products amplifying the nanoluciferase gene from *T. vaginalis* genomic DNA preparations. Highlighted (and arrows) are residues G50C and A54T which are the site of the two predicted modifications for repair of the stop codon (A54T) and oligo-modification confirmatory mutation (G50C). Immunoblot analyses in Fig. 4C and D were imaged using a Bio-Rad Gel Doc and ImageLab software. Full length blots/gels are presented in Supplementary Figure S5.

To further explore the ability to utilize CRISPR/Cas9 in *T. vaginalis*, we co-nucleofected parasites with pCas9-gRNA(*nluc*), pMN::*nluc(stop)* and either the 50 bp or 125 bp annealed repair oligos and selected for parasites containing both the pCas9-gRNA(*nluc*) (puromycin selection) and pMN::*nluc(stop)* (G418 selection) plasmids (Fig. 4A). Assaying for nanoluciferase signal 24 hours post-nucleofection, prior to drug selection, yielded a low overall signal compared to previous nucleofections (Fig. 4B). This is likely the result of requiring the same parasite to acquire all three DNAs to allow Cas9-directed repair. We also found that the 50 bp annealed oligo treatment did not work effectively, compared to previous experiments using parasites preselected to contain pMN::*nluc(stop)* target. Conversely, treatment with the 125 bp annealed oligos yielded a ~six-fold increase in nanoluciferase signal over background (water).

We then subjected the nucleofected parasites to selection with G418 and puromycin for either 7 or 14 days and tested for nanoluciferase activity. The amount of nanoluciferase signal observed was very high for parasites nucleofected with the 125 bp repair oligos (Fig. 4C). This signal did not increase between the 7 and 14 day measurements. To confirm expression of intact nanoluciferase protein at 14 days, total protein was extracted and analyzed by immunoblot analysis for the presence of repaired nanoluciferase-2xHA. We observed expression of the protein in parasites nucleofected with all three DNAs (pCas9-gRNA(*nluc*), pMN::*nluc(stop)* and the 125 bp annealed repair oligos), but not in parasites where the pCas9-gRNA (*nluc*) was replaced with an empty vector (pMPAC::EV) (Fig. 4C, inset), consistent with the nanoluciferase activity assay. These data show that the repair of the dead luciferase was dependent on the presence of both essential components of the CRISPR/Cas9 system: the Cas9 and gRNA.

To confirm the repair of nanoluciferase by directly examining the DNA sequence of the luciferase gene, we cloned parasites from a selected population demonstrated to express the enzyme by activity and immunoblot assays, using limiting dilution. We then assayed 79 clones for nanoluciferase activity and observed that 91% (72 clones) gave signal above background, while 9% (7 clones) were below a cutoff of 30,000 relative luminescence units. The high percentage of clones expressing nanoluciferase indicates that the majority of parasites in the culture were expressing a repaired nanoluciferase gene. To support this observation, we collected total protein from 8 clones and performed immunoblot analysis (Fig. 4D, upper). Although varied in amount of observable signal, we were able to detect nanoluciferase signal in all 8 clones. We further PCR amplified and then sequenced the *nluc* gene from total gDNA extracts of 8 clones (Fig. 4D, lower). In all 8 clones, the codon 17 contained a mixed population of guanine (GGA, glycine) and cytosine (GCA, alanine) sequence. Additionally, without exception, codon 18 contained a mixed population of thymine (TAT, tyrosine) and adenine (TAA, ochre/stop). The presence of a mixed population of *nluc(stop)* (GGATAA or GCATAA) and *nluc(repaired)* (GGATAT or GCATAT) sequences in the examined clones is expected. Our previous study indicates that ~15–20 episomes are maintained per *T. vaginalis* cell^53^, hence CRISPR/Cas9-mediated repair may not reach every single plasmid copy within a cell. In fact, repair of a single modified *nluc(stop)* gene would likely be sufficient to be detectable by DNA sequencing and to produce enough luciferase activity for detection using the sensitive NanoGlo assay (Figure S2). Together, the data presented clearly demonstrate that the expression of Cas9 & gRNAs in *T. vaginalis* can mediate gene repair via the CRISPR/Cas9 pathway.

### CRISPR knockout of the Ferredoxin-1 and Mif genes from the *T. vaginalis* genome

**Figure 5:**
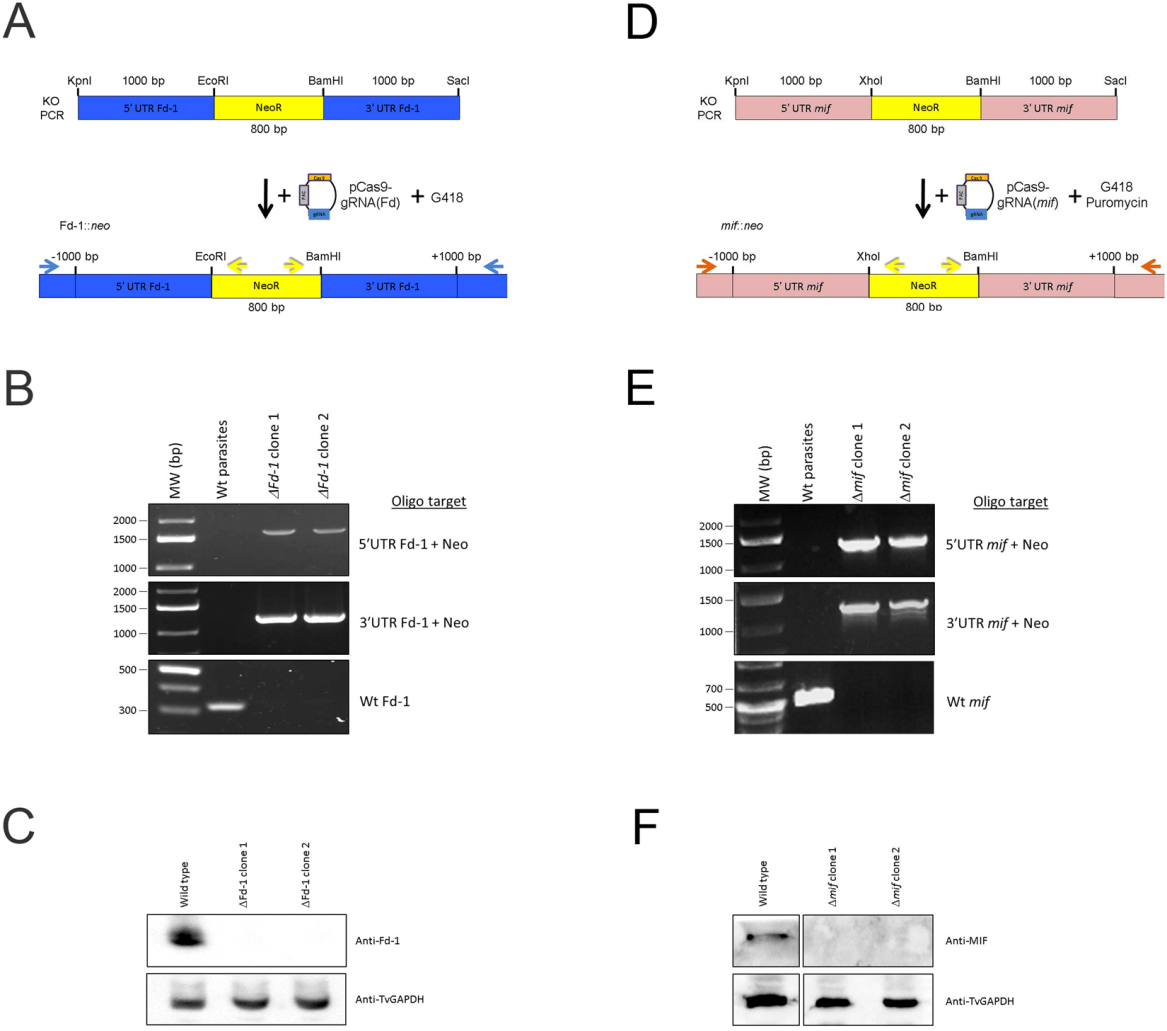
CRISPR-directed KO of ferredoxin-1 and *mif*. (**A**) Cartoon representation of the KO scheme using nucleofection of parasites with a linearized neo resistance gene flanked by the ferredoxin-1 UTRs and a plasmid containing Cas9 and Fd gRNA (pCas9-gRNA(*Fd*)) followed by selection for G418-resistance (image not to scale). The resulting parasites were then screened for the presence of the KO and wt alleles using sequence-specific primers (horizontal arrows). (**B**) PCR analysis of G418-resistant clones for the presence of the neo resistance gene in the ferredoxin-1 locus. PCR screens utilized primers specific to the neo resistance gene (yellow arrows in 5 A) combined with primers specific to the ferredoxin-1 locus (blue arrows in 5 A) in regions outside the region present in the original KO PCR introduced by nucleofection. The 5′ end PCR screen (upper panel) predicts a product of 1665 bp and the 3′ PCR screen (middle panel) predicts a product of 1236 bp if the neo gene is present in the ferredoxin-1 locus. Lowest panel: PCR analysis of the ferredoxin-1 gene in clones. Amplification utilized primers specific to the wild-type ferredoxin-1 (yielding a 324 bp product, if present). The PCR products for the different panels were run in parallel from reactions on 100 ng of the indicated genomic DNA extracts and resolved on separate agarose gels then imaged using a Bio-Rad Gel Doc and ImageLab software. (**C**) Immunoblot protein analysis of the clones yielding positive PCR products for KO in Fig. 5B. Total protein extracts were resolved by SDS-PAGE and immunoblotted using anti-Ferredoxin-1 and TvGAPDH antibodies. All immunoblots contained equal amounts of the same experimental samples and were analyzed in parallel using a Bio-Rad Gel Doc and ImageLab software. (**D**) Cartoon representation of the KO scheme using nucleofection of parasites with a linearized neo resistance (NeoR) gene flanked by the *mif* UTRs and a plasmid containing Cas9 and *mif* gRNAs (pCas9-gRNA(*mif*)), followed by selection for G418- and puromycin-resistance (image not to scale). The resulting parasites were then screened for the presence of the KO and wt alleles using sequence-specific primers (horizontal arrows). (**E**) PCR analysis of G418 and puromycin-resistant clones for the presence of the neo resistance gene in the *mif* locus. PCR screens utilized primers specific to the neo resistance gene (yellow arrows in 5D) combined with primers specific to the *mif* locus (orange arrows in 5D) in regions outside the region present in the original KO PCR introduced by nucleofection. The 5′ end PCR screen (upper panel) predicts a product of 1290 bp and the 3′ PCR screen (middle panel) predicts a product of 1220 bp if the neo gene is present in the *mif* locus. Lowest panel: PCR analysis of the *mif* gene in clones. Amplification utilized primers specific to the wild-type *mif* gene (yielding a 500 bp product, if present). The PCR products for the different panels were run in parallel from reactions on 100 ng of the indicated genomic DNA extracts and resolved on separate agarose gels then imaged using a Bio-Rad Gel Doc and ImageLab software. (**F**) Immunoblot protein analysis of the clones yielding positive PCR products for KO in Fig. 5E. Total protein extracts were resolved by SDS-PAGE and immunoblotted using anti-MIF and TvGAPDH antibodies. All immunoblots contained equal amounts of the same experimental samples and were analyzed in parallel using a Bio-Rad Gel Doc and ImageLab software. Full length blots/gels are presented in Supplementary Figure S5.

To test whether the CRISPR/Cas9 system we have established is capable of knocking out a gene in the complex *T. vaginalis* genome, we first attempted to knockout the *ferredoxin-1* (Fd-1) gene previously shown to be non-essential to the parasite^29,67^. A neomycin resistance (NeoR) gene flanked by ~1000 bp of the 5′ and 3′ UTRs of Fd-1 was constructed (Fig. 5A). Parasites were co-nucleofected with either the pMPAC::EV or the pCas9-gRNA(Fd) plasmid that expresses a gRNA targeting the 5′ end of the gene and an ~2800 bp PCR product containing the NeoR genes flanked by Fd-1 gene 5′ and 3′ UTRs (Fig. 5A). After selection for parasites resistant to G418, parasites were screened for the presence of the NeoR gene in the Fd-1 locus, using PCR and primers that sit just outside the Fd-1 UTRs included in the ~2800 bp PCR product. Positive amplicons of expected size were observed in the pCas9-gRNA(Fd) nucleofected parasites, while the pMPAC::EV parasites did not grow as expected. We then obtained cloned parasites from a NeoR positive population using limited-dilution cloning. Two clones (2–2 G4 “*ΔFd-1* clone 1” and 2-1 D9 “*ΔFd-1* clone 2”) were randomly selected and tested for the presence of the NeoR gene as well as the wild-type Fd-1 gene (Fig. 5B). Confirmation of the sequence of all PCR products clearly demonstrates that both clones contain the NeoR gene in the Fd-1 locus and to also lack the endogenous Fd-1 gene (Fig. 5B). Immunoblot analysis using an antibody that detects Fd-1^67^ also confirmed the loss of Fd-1 protein in both clones (Fig. 5C).

To further test the CRISPR/Cas9 system, we attempted to knockout the single copy *mif* gene, which codes for a homologue of the human macrophage migration inhibitory factor (MIF) and was recently reported to affect host cell physiology^68^. A neomycin resistance (NeoR) gene flanked by ~1000 bp of the 5′ and 3′ UTRs of *mif* was constructed (Fig. 5D). To illustrate the ability to use CRISPR/Cas9 in multiple *T. vaginalis* strains, the B7RC2 strain^22^ was used. Parasites were co-nucleofected with the pCas9-gRNA(*mif*) plasmid expressing a gRNA targeting the 5′ end of the gene and an ~2800 bp PCR product containing the NeoR gene flanked by *mif* gene 5′ and 3′ UTRs (Fig. 5D). After selection for parasites resistant to G418 and puromycin, parasites were screened for the presence of the NeoR gene in the *mif* locus, using PCR and primers that sit just outside the *mif* UTRs included in the ~2800 bp PCR product. Positive amplicons of expected size were observed in the pCas9-gRNA(*mif*) nucleofected parasites (Fig. 5E). Control reactions wherein parasites were nucleofected with only the linear KO PCR product survived G418 selection, however no positive PCR signal was observed for the NeoR gene in the *mif* locus (Supplementary Figure S4). We further examined these control parasites lacking the pCas9-gRNA(*mif*) plasmid by screening 800 cells, split into 40 subpopulations of 20 parasites each to directly test whether the NeoR gene was in the *mif* locus. No PCR product was detected in any of the 40 subpopulations (Supplementary Figure S4). This is in contrast with obtaining positive PCR signal for NeoR integration at the *mif* locus in 39/40 subpopulations of the parasites nucleofected with both the pCas9-gRNA(*mif*) plasmid and the linear KO PCR product. To confirm that the *mif* gene was knocked out in these NeoR PCR positive populations, parasites were cloned using limited-dilution cloning. Two clones (“*Δmif* clone 1” and “*Δmif* clone 2”) were randomly selected and tested for the presence of the NeoR gene in the *mif* locus and the absence of the wild-type *mif* gene (Fig. 5E). Confirmation of the sequence of all PCR products clearly demonstrated that both clones contain the NeoR gene in the *mif* locus and also lack the endogenous *mif* gene (Fig. 5E). Immunoblot analysis using an antibody that detects MIF likewise confirmed the loss of the MIF protein in both clones (Fig. 5F). Together, our findings demonstrate that CRISPR/Cas9 can be used to disrupt gene sequences in the *T. vaginalis* genome by a homology-directed repair pathway.

## Discussion

Here we describe the development of a system to achieve CRISPR/Cas9 gene modification and gene knockout in *Trichomonas vaginalis*. Three technical advances made this possible. One was to decrease the toxicity of Cas9 when expressed in *T. vaginalis* by using FKBP-DD/Shield-1 regulated expression of the endonuclease. The second was to increase transfection efficiency using nucleofection. These together, with the use of a sensitive nanoluciferase reporter assay to detect homology directed repair of a premature stop codon back to wild-type firstly described by Streipen and colleagues^47,69^, provided a sensitive assay to facilitate identifying optimal nucleofection parameters. Finally, we were able to utilize the strong RNA polymerase III *T. vaginalis* U6 promoter to drive transcription of a sequence-customizable gRNA. This, the first report of CRISPR/Cas9 gene modification in *T. vaginalis*, will greatly enhance the ability to test gene function in this pathogen.

It has been previously reported that expression of Cas9 in various organisms leads to cell growth issues^43^. We found that initial attempts at plasmid-based Cas9 expression by transfection and selection of *T. vaginalis* repeatedly failed. Different reports have addressed decreased cell growth by transient transfection of *cas9-gRNA* from plasmids or transfecting with Cas9 mRNA or holoenzymes^70–72^. We chose to regulate Cas9 expression at the protein through fusion to the FKBP-destabilization domain (FKBP-DD), which by default sends proteins for proteolytic degradation. In the presence of a stabilizing ligand Shield-1, the protein accumulates^54,55^. We found that this method allowed the regulation of cellular Cas9 levels in *T. vaginalis*, which in turn yielded viable parasites expressing sufficient, but not toxic, levels of Cas9.

Testing the biological role of genes in *T. vaginalis* has previously been severely compromised by limited technologies to alter the highly, repetitive genome of this parasite^52^. Only two previous reports have successfully knocked out *T. vaginalis* genes^28,29^ and knockdown of gene expression is limited to four reports^30–33^. Lacking the ability to knockout gene expression, researchers have primarily relied on episomal over-expression of gene variants to study protein function.

One reason that homologous gene replacement has been hampered in *T. vaginalis* is the poor transfection efficiency achieved with standard electroporation. Here, using nucleofection^64,73^, we have greatly enhanced the transfection efficiency by 20-fold or greater as compared to that achieved using electroporation method. This enhancement of transfection efficiency has enhanced both the probability of successful modification of genes and the ability to detect the modifications by sensitive techniques such as nanoluciferase activity assays or PCR. Thus, developing successful nucleofection methods allowing sensitive screening and selection for gene modifications was key to our ability to achieve and monitor CRISPR/Cas9 modification of *T. vaginalis* genes.

Our analyses of the *T. vaginalis* genome for homologs of NHEJ pathway components indicated that this pathway is absent or highly divergent from other organisms. Based on this observation, we focused on homology-directed repair pathways to establish the use of CRISPR/Cas9 in this parasite. We were able to detect CRISPR activity using the sensitive technique of early termination mutation repair first used to demonstrate CRISPR in *Crytosporidium parvum* ^47^. Using a 125 bp repair template of annealed oligonucleotides with flanks on both sides of the CRISPR cut site, we were able to repair an early termination codon restoring nanoluciferase activity as demonstrated by enzyme activity assays, immunoblot analysis and DNA sequencing. The repair template makes two specific point mutations both changing the stop codon to a tyrosine and a proximal glycine to alanine. The nanoluciferase repair assay demonstrates that small oligonucleotides are sufficient to modify multi-target episomally maintained genes and thus the length of a homology template may not have to be large. Repair is due to homology-directed repair (HDR) using the oligonucleotide template, as opposed to NHEJ activity. Notably, in the absence of the HDR template, we did not observe signal of repaired nanoluciferase above background signal, consistent with the lack of NHEJ-mediated repair.

Nucleofection of pre-selected pMN::*nluc(stop)* parasites with pCas9-gRNA(*nluc*) and repair oligos resulted in an increase of nanoluciferase activity. This varied based on concentration of oligos with 100 μg working more effectively than 10 μg. Transient co-nucleofection of all DNA components (pMN::*nluc(stop)*, pCas9-gRNA(*nluc*) and repair oligos) resulted in lower nanoluciferase signals, however, this could be overcome using drug selection to enrich for parasite populations containing DNAs required for CRISPR/Cas9-mediated repair. Using drug selection and cell cloning by limited dilution, we found that 91% of the clones had nanoluciferase activity above background. DNA sequencing of the clones confirmed the modification of the *nluc(stop)* to *nluc(repaired)*, demonstrating CRISPR-Cas9 mediated repair of the *nluc(stop)* gene.

We have also demonstrated the ability to use CRISPR-Cas9 mediated homology-repair to eliminate two genes (*ferredoxin-1* and *mif*) from the genome of *T. vaginalis*. CRISPR-mediated gene knock-out was achieved in two different *T. vaginalis* strains. The methods we have developed to increase transfection efficiency for both plasmids and KO cassettes and to direct CRISPR/Cas9 gene modification will greatly increase the success rate of gene knockouts in this parasite. The expanded toolkit for molecular modification of the *T. vaginalis* genome will significantly enhance the ability to assess gene function in this medically-important pathogen.

## Methods

### Parasite culture

Parasites (*T. vaginalis* strain B7RC2^22^ and MSA 1103^74^) were maintained by daily passage in modified TYM media^75^ supplemented with 10% heat-inactivated horse serum (Sigma), 10 U/ml penicillin/10 μg/ml streptomycin (Invitrogen), 180 μM ferrous ammonium sulfate and 28 μM sulfosalicylic acid (completed TYM media). To select for the pMasterPAC (pMPAC)^53,76^ or pMasterNEO (pMN)^22^ plasmids, puromycin (AG scientific) or G418 (Invitrogen) was added to cultures to a final concentration of 30 μg/ml and 100 μg/ml, respectively.

### Plasmid constructs

Plasmid pMN::*cas9* was created by PCR amplification of the human codon optimized *cas9* gene of plasmid pMJ920^56^ using primers For-hcas9-SacII and Rev-hcas9-Bam. All oligos used in this study are listed in Supplemental Table 1. The resulting hcas9 product was digested with SacII/BamHI and ligated to pMN::*fkbp*. The resulting construct was then PCR amplified using primers For-dS-HuCas9 and Rev-MN3UTR to silently mutate an internal SacI sequence. This product was then used in megaprimer PCR with primer For-MN-SacXho and subsequently digested with SacI/BamHI and ligated into pMN::*fkbp* to produce pMN::*fkbp-cas9*. To create the pMPAC::*fkbp-cas9* construct, pMN::*fkbp-*cas9 was digested with SacI/BamHI and the product cloned into the pMPAC-empty vector plasmid (pMPAC::EV). The gRNA cassette was constructed by PCR amplification of a synthetic gene construct (Table S1) containing the U6 seed region and gRNA scaffold flanked upstream by the 360 bp 5′ of the U6 start nucleotide and downstream with 37 bp of the 3′ UTR of the U6 gene using primer U6_SacI_F1 and downstream 3′ UTR with primer U6_SacI_R1. The resulting product was then digested with SacI and ligated to the unique SacI site in pMasterNEO^53^. The *nluc* gRNA was constructed by megaprimer amplification of the gRNA scaffold using primers For-Nluc-gRNA and U6_SacI_R1 and then used in a megaprimer PCR with U6_SacI_F1. The resulting PCR product was digested with SacI and ligated to pMPAC::*fkbp*-*cas9*. The final construct was termed to pMPAC::*fkbp-cas9-gRNA(nluc)*, abbreviated as pCas9-gRNA(*nluc*).

Construction of nanoluciferase plasmids utilized codon-optimized plasmid templates of *nluc* (wt) and *nluc* (Y18ochre, “dead”/stop) from the Nluc-Neo and Dead Nluc-Neo plasmids (a generous gift from the Boris Striepen laboratory, University of Georgia)^47^, respectively. For each *nluc* variant, a megaprimer PCR was performed utilizing primers For-MN-SacXho and Rev-Nluc-Nde to amplify the 5′ UTR of a-SCS on pMasterNEO with a portion of the 5′ end of the *nluc* gene. The resulting products were gel-purified and used with primer Rev-Nluc-Kpn to amplify the remainder of *nluc* and *nluc(stop)*. The PCR products were then digested with SacI/KpnI and ligated to pMasterNEO such that the *nluc* (or repaired) sequence would by fused to a 2xHA epitope tag when translated. These plasmids were termed pMN::*nluc* and pMN::*nluc(stop)*.

For construction of the pCas9-gRNA construct targeting the *ferredoxin-1* gene (TVAG_003900), the 20 nt seed region of pMPAC::*fkbp-cas9-gRNA(nluc)* was replaced after the adenosine reside start with 19 nucleotides of the *ferredoxin-1* gene (residues 5–23, 5′ - TCT CTC AAG TTT GCC GCT T) which lie 5′ upstream of a TGG PAM sequence. This was constructed by PCR amplification with primers For-gRNA-Fer-1 and U6_SacI_R1. The resulting PCR product was purified and used in a second PCR with primer U6_SacI_F1, followed by digestion with SacI and cloning into pMPAC::*fkbp-cas9-gRNA(nluc)*. The resulting plasmid was termed pCas9-gRNA*(Fd)*. For construction of the pCas9-gRNA construct targeting the *mif* gene (TVAG_219770), a dual gRNA was constructed. For each of the gRNAs the 20 nt seed region of pMPAC::*fkbp-cas9-gRNA(nluc)* was replaced after the adenosine reside start with 19 nucleotides of the *mif* gene. The *mif* sequences “g300” (5′ - CAA AGA GTG CTG AGG ACT G) and “g301” (5′ -CCA AAG AGTGCT GAG GAC T) were used to construct 19 nucleotides after the A in the seed region. Briefly, “g300” gRNA was constructed using PCR primers For-gRNA-g300 + U6-KpnI-R. The resulting product was purified and used in a second PCR with primer U6_SacI_F1. For “g301” gRNA was constructed using PCR primers For-gRNA-g301 + U6_SacI_R1. The resulting product was purified and used in a second PCR with primer U6-Kpn-F. Both PCR products were digested with KpnI, ligated together and gel purified. The resulting ligated product was then digested with SacI and ligated into pMPAC::*fkbp-cas9-gRNA(nluc)* producing the plasmid pMPAC::*fkbp-cas9-gRNA(mif)*.

Construction of the ferredoxin KO cassette utilized 1000 bp upstream of the *ferredoxin-1* start codon and 1000 bp downstream of the stop codon. Briefly, the *neo* resistance gene was PCR amplified using primer For-Neo-Eco and Rev-Neo-Bam. The resulting product was cloned into pSC-A (Stratagene) to produce the plasmid pSC-A::*neo*. PCR amplification of the 5′ UTR of ferredoxin utilized For-Fer-Kpn and Rev-Fer-Eco. The product was then digested with KpnI and EcoRI and cloned into the pSC-A::*neo* plasmid resulting in plasmid pSC-A::5′*UTR-Fd-neo*. Amplification of the 3′ UTR of ferredoxin utilized For-Fer-Bam and Rev-Fer-Sac. The resulting product was digested with BamHI and SacI and cloned into the pSC-A::5′*UTR-Fd-neo* plasmid to produce plasmid pSC-A::5′*UTR-Fd-neo-3*′*UTR-Fd* (termed pKO-Fd).

Construction of the *mif* KO cassette utilized the same method used to generate the ferredoxin KO cassette except using *mif* gene-specific primers. Briefly, the 1000 bp upstream of the *mif* start codon and downstream of the stop codon were ligated to the NeoR gene. PCR amplification of the 1000 bp 5′ UTR upstream of the *mif* start codon utilized primers For-MIF-Kpn and Rev-MIF-Xho. The resulting product was digested with KpnI and XhoI and ligated to the pSC-A::*neo* plasmid resulting in plasmid pSC-A:: 5′*UTR-mif-neo*. PCR amplification of the 1000 bp 3′ UTR downstream of the *mif* stop codon utilized primers For-MIF-Bam and Rev-MIF-Sac. The resulting products were digested with BamHI and SacI and ligated to pSC-A::5′*UTR-mif-neo* to produce plasmid pSC-A::5′*UTR-mif-neo-3*′*UTR-mif* (termed pKO-*mif*).

### Repair oligos/PCR

Repair oligos were constructed to target mutations in the *nluc(stop)* gene (Operon). Annealing of the 125 nt repair oligos utilized Repair-oligo-F and Repair-oligo-R. Annealing of the 50 nt repair oligos utilized Repair-oligo-F50 and Repair oligo-R50. An equal concentration of each repair oligo (200 μg) was annealed in 1x NEB T4 ligase buffer to create a stock of anneal oligos. For PCR amplification of the 125 bp repair sequence, primers For-125bp and Rev-125bp were used on the annealed 125 bp oligos above. For full length PCR of *nluc*, the digested (SacI/KpnI) and gel purified template of pMN::*nluc* was used as a PCR template using primers For-pMN-5UTR and Rev-Nluc-Kpn. The resulting product was treated with DpnI and then gel purified and amplified using the same primers. The final PCR product was precipitated, resuspended and quantified.

PCR amplification of the *ferredoxin-1::neo* knockout utilized the primers For-Fer-Kpn and Rev-Fer-Sac on a gel-purified product from a KpnI and SacI digestion of pKO-Fd. PCR amplification of the *mif::*neo knockout utilized the same method described above with *mif-*specific primers For-MIF-Kpn and Rev-MIF-Sac. The resulting PCR products were treated with DpnI and confirmed to be free of template plasmid. The final KO PCR product was precipitated, resuspended and quantified.

### Protein and Immunoblot analysis

For Shield-1 (Clontech) titration, parasites were seeded at 1 × 10^5^ cells/ml and treated with the indicated concentrations for 24 hours. For all immunoblot analyses, total protein was extracted and analyzed by SDS-PAGE using equal amount of soluble protein (10 μg). Protein was then transferred to a membrane which was subsequently incubated with an anti-Cas9 antibody (Clontech). For TvGAPDH detection, the membrane was incubated with an anti-TvGAPDH antibody (Cocalico Biologicals). Images were captured and analyzed by Bio Rad Gel Doc and ImageLab software (v. 5.1, Bio Rad). Resulting values were normalized to TvGAPDH signal and presented as the average +/− the standard deviation from two independent analyses. For detection of nanoluciferase-2xHA protein, total protein was extracted from parasites, resolved by SDS-PAGE, transferred to a PVDF membrane and incubated with an anti-HA antibody (Covance). For immunoblot analysis of the Ferredoxin-1 and MIF KO samples, total soluble protein from parasite samples was resolved by SDS-PAGE, transferred to a membrane and incubated with an anti-Ferredoxin-1^67^, anti-MIF^68^ and anti-TvGAPDH (Cocalico Biologicals) antibodies. Uncropped images for the corresponding immunoblots are included in Figure S5.

### Nanoluciferase activity assay

A total of 1 × 10^6^ parasites were harvested per reaction (in duplicate), washed in ice-cold 1x PBS with 5% sucrose (w/v) and resuspended at a concentration of 1 × 10^6^ cells/100 μl in the same PBS/sucrose solution. Each 100 μl aliquot was mixed with 100 μl of completed NanoGlo luciferase assay reagent (Promega) and transferred to round bottomed white walled 96-well plates (Costar). Luciferase activity was analyzed by measurement with a Victor 3 model 1420 plate reader at 460 nm. Relative luminescence signal was measured in duplicate or triplicate for each assay and the average signal calculated +/− standard deviation.

### Transfection of T. vaginalis using nucleofection and electroporation

Parasites were collected by centrifugation and resuspended in 100 μl T-cell, Parasite-1 or Parasite-2 buffers (Lonza) and the indicated amounts of DNA and parasites (1 × 10^7^ or 5 × 10^7^). Typically, 10 μg plasmid(s) and/or 100 μg of anneal oligos or PCR product was added. After five minutes of incubation at room temperature, parasites were nucleofected using an Amaxa nucleofector using the programs U-033, D-023 or X-100. For parasite electroporation, 5 × 10^7^ parasites were resuspended in 100 μl of completed TYM media and placed into electrocuvettes with the indicated DNA. Parasites were then electroporated using a single pulse of 350 V with 975 μF capacitance using a Bio Rad Gene Pulser II^53^. For both nucleofection and electroporation parasites were immediately resuspended into 50 ml of fresh completed TYM media. During recovery, parasites were treated with 1 μM Shield-1 where indicated.

Knockout of the *ferredoxin-1* gene (TVAG_003900) in the MSA 1103 *T. vaginalis* strain^74^ utilized the nucleofection conditions (U-033 and T-cell buffer) with the substitution of pCas9-gRNA*(Fd)* or pMPAC::EV and 50 μg of KO PCR product in duplicate. Parasites were recovered in completed TYM media for four hours and then selected for resistance to 50 μg/ml of G418. After an additional 16 hours, the parasites were harvested and resuspended in fresh completed TYM media and 100 μg/ml G418. After outgrowth from selection to 1 × 10^6^ parasites/ml, genomic DNA was extracted from ~1.5 × 10^7^ parasites samples and PCR screening was done for the presence of the KO allele at the *ferredoxin-1* locus (see below). The cultures with signal positive for *ferredoxin-1* disruption were diluted and re-screened using limiting dilution until clones were obtained. The clones were screened for the presence of the *neo* gene at the ferredoxin locus as well as for the presence of the wild type *ferredoxin-1* gene. All PCRs were performed on 100 ng of purified genomic DNA and utilized NEB Phusion polymerase, per the manufacturers’ instructions. Screening for the wild-type gene used primers For-003900-1 and Rev-003900-1 (PCR cycle: 95 °C- 30 sec, 60 °C- 30 sec, 72 °C- 90 sec). Screening for the 5′ end of the *ferredoxin-1::neo* KO locus used primers For-003900-5UTR-1 and Rev-Neo-Int3 (PCR cycle: 95 °C- 30 sec, 60 °C- 30 sec, 72 °C- 90 sec). Screening for the 3′ end of the *ferredoxin-1::neo* KO locus used primers For-Neo-Int2 and Rev-003900-3UTR-2 (PCR cycle: 95 °C- 30 sec, 64 °C- 30 sec, 72 °C- 90 sec). All PCR products were confirmed by DNA sequencing.

Knockout of the *mif* gene (TVAG_219770) in *T. vaginalis* strain B7RC2^22^ utilized nucleofection conditions described for the knockout of ferredoxin-1 above, except the D-023 program and V-kit buffer were utilizied, and pCas9-gRNA*(mif)* or water (for the negative control) and 100 μg of KO PCR product was used. B7RC2 parasites were recovered in completed TYM media for 24 hours and selected for resistance to 100 μg/ml of G418, and 30 μg/ml of puromycin for the parasites that received pCas9-gRNA(*mif*). Negative control parasites that did not receive pCas9-gRNA(*mif*) were only selected with 100 μg/ml of G418. After an additional 24 hours, the parasites that received pCas9-gRNA(*mif*) were re-dosed with the 100 μg/ml G418, and 30 μg/ml of puromycin and control parasites were re-dosed with just 100 μg/ml G418. Drug selected parasites were then sub-populated into 20 cells per well in a 96-well plate. When the parasites reached ~1 × 10^7^ parasites/ml, genomic DNA was prepared for PCR screening. Initial screening was done utilizing the 5′ end of the *mif::neo* KO with primers For-219770-5UTR-1 and Rev-Neo-Int4 (PCR cycle: 95 °C- 30 sec, 55 °C- 30 sec, 72 °C- 90 sec). A subpopulation with the correct Neo replacement based on 5′ UTR PCR analyses was cloned and then screened for the presence of the NeoR gene in the *mif* locus using both the 5′ primer described above and the 3′ primer For-Neo-Int3 and Rev-219770-3UTR-1 (PCR cycle: 95 °C- 30 sec, 55 °C- 30 sec, 72 °C- 90 sec). Screening for the wild-type *mif* gene used primers For-219770-1 and Rev-219770-1 (PCR cycle: 95 °C- 30 sec, 55 °C- 30 sec, 72 °C- 30 sec). All PCR products were confirmed by DNA sequencing.

### RT-PCR analysis of gRNA expression

Total RNA was isolated from ~1.5 × 10^7^ parasites using the Direct-zol RNA Miniprep kit (Zymo) and then fractionated into small RNAs using the RNA Clean and Concentrator kit (Zymo). Small RNAs were further treated with the *TURBO DNA-free* kit (Ambion). Poly-A tails were then added using the Poly(A) polymerase tailing Kit (Ambion). Small RNAs were quantified and cDNA created by utilizing an equivalent amount of RNA in the Superscript III first strand synthesis system for RT-PCR (Invitrogen) using the provided oligo-dT_20_ primer. All PCRs were performed on one microliter of the resulting cDNA and utilized NEB Phusion polymerase, per the manufacturers’ instructions. For the gRNA, primers For-gRNA and Rev-gRNA were used to detect the gRNA and primers For-U6 and Rev-U6 were used to detect the U6 snRNA (for both, the PCR cycle: 98 °C- 10 sec, 55 °C- 20 sec, 72 °C- 8 sec). All samples were resolved on agarose-TBE gels, stained with ethidium bromide and imaged by Bio-Rad Gel Doc, as above.

### Data Availability

All data generated or analysed during this study are included in this published article (and its Supplementary Information files).

## Electronic supplementary material

Supplemental Dataset
