# Supplementary material for: CRISPR/Cas9-mediated gene modification and gene knock out in the human-infective parasite Trichomonas vaginalis
Source: Sci Rep. 2018 Jan 10;8:270. doi: 10.1038/s41598-017-18442-3 (PMC5762654; doi:10.1038/s41598-017-18442-3)
Supplement: Supplementary file 1 — Supplemental Dataset [file 41598_2017_18442_MOESM1_ESM.docx]

**Revised Supplemental Material**

**CRISPR/Cas9-mediated gene modification and gene knock out in the human-infective parasite *Trichomonas vaginalis***

Brian D. Janssen^1^, Yi-Pei Chen^1,2^, Brenda M. Molgora^1,2^, Shuqi E. Wang^3^, Augusto Simoes-Barbosa^3^, & Patricia J. Johnson^1,2,*^

^1^ Department of Microbiology, Immunology & Molecular Genetics, ^2^Molecular Biology Institute, University of California, Los Angeles, California, USA, ^3^School of Biological Sciences, University of Auckland, New Zealand

**Supplemental Material**

**Supplemental Figure Legends**

**Figure S1**: Comparison of nucleofection vs. electroporation. Parasites were nucleofected or electroporated with 10 μg of pMN::*nluc* (or mock transfected). Nucleofection utilized T-cell, Parasite-1 or Parasite-2 buffers (Lonza). Parasites were then transfected with program U-033, X-100 or D-023 (Amaxa). For electroporation, parasites were transfected with either 10 or 50 μg of pMN::*nluc* in completed TYM media. In all cases, parasites were recovered, counted and luminescence measured after 24 hours of recovery in completed TYM media. Total luminescence was calculated after normalization to % survival rate compared to mock transfection for each buffer condition.

**Figure S2**: Detection of luminescence from different numbers of parasites pre-selected for pMN::nluc. Luminescence was measured from parasites pre-selected for the pMN::nluc plasmid. Parasites were then counted and parasite numbers adjusted to the indicated number and luminescence measured. The graph represented the average luminescence value +/- standard deviation from two independent measurements per parasite counts.

**Figure S3**: Comparison of transient nucleofection of pMN::nluc to parasites pre-selected for plasmid. Parasites were transiently nucleofected and nanoluciferase activity measured at 24 hours post-nucleofection versus PBS and pre-selected pMN::*nluc* and pMN::*nluc(stop)* control parasites. For pMN::*nluc* nucleofection, a ~20-fold increase in parasite numbers from the transiently nucleofected samples is necessary to achieve signal comparable to the pre-selected parasites. Equivalent numbers of parasites (1x10^6^) were assayed for nanoluciferase activity and the graph represents the average relative luminescence value +/- standard deviation of two attempts per condition.

**Figure S4: PCR screen of parasites transfected with water + *mif::neo* KO PCR.** Representative image of 5’ UTR PCR screen of subpopulations (20 parasites/ subpopulation) of parasites nucleofected *mif::neo* KO linear PCR product without the Cas9-gRNA(mif) plasmid and subjected to selection with G418. Water replaced the Cas9-gRNA (mif) plasmid. The PCR screen for *neo* in the *mif* locus (a product of 1290 bp) utilized oligonucleotides For-219770-5UTR-1 and Rev-Neo-Int4. In total 1000 parasites we screened and all subpopulations lacked detectable PCR products for the KO allele. “Δ*mif* clone” shows positive control PCR reaction on a KO clone that contains *neo* in the *mif* locus. Image is uncropped.

**Figure S5**. Un-cropped images relating to the figures. Dotted boxes indicate cropped portion used in respective figures.


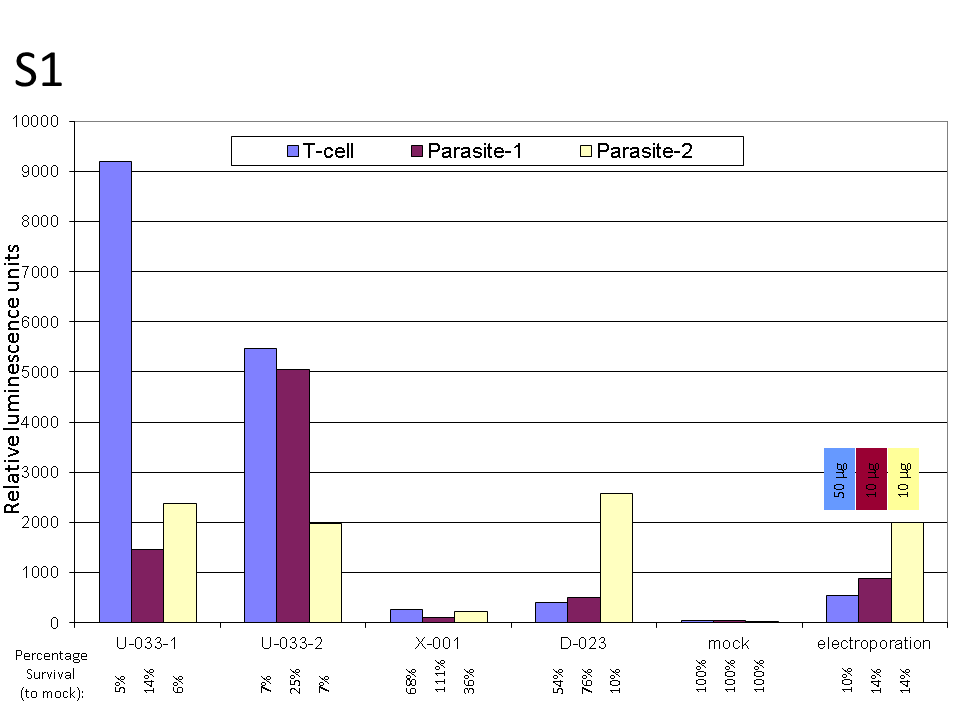


**Supplementary Figure S1: Comparison of nucleofection vs. electroporation**. Parasites were nucleofected or electroporated with 10 μg of pMN::*nluc* (or mock transfected). Nucleofection utilized T-cell, Parasite-1 or Parasite-2 buffers (Lonza). Parasites were then transfected with program U-033, X-100 or D-023. For electroporation, parasites were transfected with either 10 or 50 μg of pMN::*nluc*. In all cases, parasites were recovered, counted and luminescence measured after 24 hours of recovery. Total luminescence was calculated after normalization to % survival rate compared to mock transfection for each buffer condition.

**Supplementary Figure S2: Detection of luminescence from different numbers of parasites pre-selected for pMN::*nluc*.** Luminescence was measured from parasites pre-selected for the pMN::*nluc* plasmid. Parasites were then counted and parasite numbers adjusted to the indicated number and luminescence measured. The graph represented the average luminescence value +/- standard deviation from two independent measurements per parasite count.

**Supplementary Figure S3: Comparison of transient and pre-selected parasites.** Comparison of transient nucleofection (+24 hours post-transfection) of pMN::*nluc* into to parasites pre-selected for presence of plasmid versus PBS and pre-selected pMN::*nluc(stop)* control parasites. For pMN::*nluc* nucleofection, a ~20-fold increase in parasite numbers from the transiently nucleofected samples is necessary to achieve signal comparable to the pre-selected parasites. Equivalent numbers of parasites (1x10^6^) were assayed for nanoluciferase activity and the graph represents the average relative luminescence value +/- standard deviation of two attempts per condition.


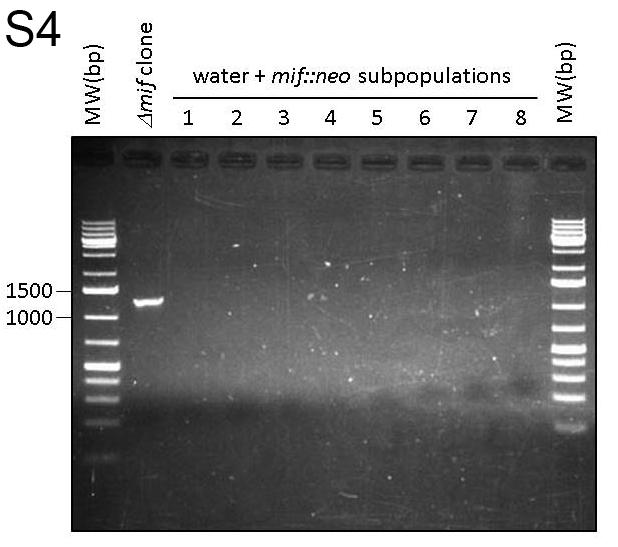


**Supplementary Figure S4: PCR screen of parasites transfected with water + *mif::neo* KO PCR.** Representative image of 5’ UTR PCR screen of subpopulations (20 parasites/ subpopulation) of parasites nucleofected *mif::neo* KO linear PCR product without the Cas9-gRNA(mif) plasmid and subjected to selection with G418. Water replaced the Cas9-gRNA (mif) plasmid. The PCR screen for *neo* in the *mif* locus (a product of 1290 bp) utilized oligonucleotides For-219770-5UTR-1 and Rev-Neo-Int4. In total 1000 parasites we screened and all subpopulations lacked detectable PCR products for the KO allele. “Δ*mif* clone” shows positive control PCR reaction on a KO clone that contains *neo* in the *mif* locus. Image is uncropped.

**

**

S5

**Supplementary Figure S5**. Un-cropped images relating to the figures. Dotted boxes indicate cropped portion used in respective figures.

**Table S1: List of oligonucleotides and gRNA(U6) synthetic gene used in this study**

| **Underlined are restriction sites or important residues** | | | |
| --- | --- | --- | --- |
| **Name** | | **Sequence** | |
| **For-hcas9-SacII** | | **5´ – AAA CCG CGG ATG GAC AAG AAG TAC AGC ATC GGC C** | |
| **Rev-hcas9-Bam** | | **5´ – GAT GGA TCC TTA GTT GCT CCC ACT ACC AAT GCC** | |
| **For-dS-HuCas9** | | **5´ - CCT GCA GAC AGT GAA GGT GGT GGA CGA ACT CGT GAA AGT GAT GGG CCG GC** | |
| **Rev-MN3UTR** | | **5´ - GCA TTT TTC TTA AGA GAG AAG CTT TCC** | |
| **For-MN-SacXho** | | **5´ - GCT GGA GCT CGA GGA ACC GAA AAT TCT TAT CTA T** | |
| **U6_SacI_F1** | | **5´ - GAG CTC ATT AAG GGT GAA TGG CTA CT** | |
| **U6_SacI_F2** | | **5´ - GAG CTC GAC TTA GAG TCT ATT GTG GA** | |
| **U6_SacI_F3** | | **5´ - GAG CTC AGG AGA AAG AGA ATT TCT GC** | |
| **U6_SacI_R1** | | **5´ - GAG CTC AAA AAA TGG GAC CTA TCC AGA** | |
| **For-Nluc-gRNA** | | **5´ - GTC AAA CAT ATT CTA TTA GAG ATT GGA GAC AAA CTG CGT TTT AGA GCT AGA AAT AGC** | |
| **Rev-Nluc-Nde** | | **5´ - CCT ACA AAA TCT TCT AAT GTA AAT ACC ATA TGA TGT GAA GTG AAC AAA GCC ATT TTA GGC** | |
| **Rev-Nluc-Kpn** | | **5´ - AAT GGT ACC GGC AAG AAT GCG TTC ACA T** | |
| **For-gRNA-Fer-1** | | **5´ - GTC AAA CAT ATT CTA TTA TCT CTC AAG TTT GCC GCT TGT TTT AGA GCT AGA AAT AGC** | |
| **For-Neo-Eco** | | **5´ - GAA TTC ATG ATT GAA CAA GAT GGA TTG CAC GC** | |
| **Rev-Neo-Bam** | | **5´ – GGA TCC TCA GAA GAA CTC GTC AAG AAG GCG** | |
| **For-Fer-Kpn** | | **5´ – AGT GGT ACC TTG TTT TTA CTT TTA TTT CG** | |
| **Rev-Fer-Eco** | | **5´ – GAA TTC TCG CTA AAG AGA AGT GAA GTA AAT ATT TTG** | |
| **For-Fer-Bam** | | **5´ – GGA TCC ATA ATT GAA AGT TTA TTA AAT TG** | |
| **Rev-Fer-Sac** | | **5´ – TTG GAG CTC TGG AAA ACA TGT CTG** | |
| **Repair-oligo-F** | | **5´ – ATCATATGGTATTTACATTAGAAGATTTTGTAGGAGATTGGAGACAAACTGCAGCATATAATTTAGATCAAGTATTAGAGCAAGGTGGAGTTTCTTCATTGTTTCAAAATTTAGGCGTTTCAGTG** | |
| **Repair-oligo-R** | | **5´ – CACTGAAACGCCTAAATTTTGAAACAATGAAGAAACTCCACCTTGCTCTAATACTTGATCTAAATTATATGCTGCAGTTTGTCTCCAATCTCCTACAAAATCTTCTAATGTAAATACCATATGAT** | |
| **Repair-oligo-F50** | | **5´ – TTG TAG GAG ATT GGA GAC AAA CTG CAG CAT ATA ATT TAG ATC AAG TAT TA** | |
| **Repair oligo-R50** | | **5´ – TAA TAC TTG ATC TAA ATT ATA TGC TGC AGT TTG TCT CCA ATC TCC TAC AA** | |
| **For-125bp** | | **5´ - ATC ATA TGG TAT TTA CAT TAG** | |
| **Rev-125bp** | | **5´ - CAC TGA AAC GCC TAA ATT TTG** | |
| **For-pMN-5UTR** | | **5´ - CCA CTT ACG CTT CAA TTA AGG** | |
| **For-003900-1** | | **5´ – TCT CTT TAG CAT ATG CTC TCT CAA GTT TGC CG** | |
| **Rev-003900-1** | | **5´ – AAT GGT ACC GAG CTC GAA AAC AGC ACC ATC G** | |
| **For-003900-5UTR-1** | | **5´ – GCG AAT ATA CAA TAA GGA GAG TAG GGG** | |
| **Rev-Neo-Int3** | | **5´ – CGG TCT TGA CAA AAA GAA CCG GGC GCC** | |
| **For-Neo-Int2** | | **5´ – GAT ATT GCT GAA GAG CTT GGC GGC G** | |
| **Rev-003900-3UTR-2** | | **5´ – GCT GCC TGC TCC AAA AAT AAA TTT CC** | |
| **For-gRNA** | | **5´ – AGC TGA GGA TAT GGC CTA GT** | |
| **Rev-gRNA** | | **5´ – AAA AGC ACC GAC TCG GAG** | |
| **For-U6** | | **5´ – AGC TGA GGA TAT GGC CTA GT** | |
| **Rev-U6** | | **5´ – CAC CTT TGA GTG  CAC AAA ATG TTT T** | |
| **For-gRNA-g300** | | **5´ - GTC AAA CAT ATT CTA TTA CAA AGA GTG CTG AGG ACT GGT TTT AGA GCT AGA AAT AGC** | |
| **For-gRNA-g301** | | **5´ - GTC AAA CAT ATT CTA TTA CCA AAG AGT GCT GAG GAC TGT TTT AGA GCT AGA AAT AGC** | |
| **U6-Kpn-F** | | **5´ – GGT ACC ATT AAG GGT GAA TGG CTA C** | |
| **U6-Kpn-R** | | **5´ – GGT ACC AAA AAA TGG GAC CTA TCC AGA** | |
| **For-MIF-Kpn** | | **5´ – GGT ACC TTT GTA TGA TGA TAG AGA ATA CAA A** | |
| **Rev-MIF-Xho** | | **5´ – CTC GAG AAA ACA AAG TGA GGT GAC TAT AAA AAT** | |
| **For-MIF-Bam** | | **5´ – GGA TCC AAT ACG GAT TTA ATT GTA ATA ATT CTT TTC** | |
| **Rev-MIF-Sac** | | **5´ – GAG CTC CCA AAG AAA CAT GCT GAA CAA** | |
| **For-219770-1** | | **5´ – AAG ATG CCG TAT CGT AAA TCA A** | |
| **Rev-219770-1** | | **5´ – CAA ATG TGT CAC CAT TGA AGC C** | |
| **For-219770-5UTR-1** | | **5´ – GCG CTA TTA TTC TTC TGT GGA CCT A** | |
| **Rev-219770-3UTR-1** | | **5´ – TGA TTA CAT GGC AGC AGC TGG** | |
| **Rev-Neo-Int4** | | **5´ – GTT CAT TCA GGG CAC CGG AC** | |
| **For-Neo-Int3** | | **5´ – GCT TTA CGG TAT CGC CGC TCC C** | |
|  | |  |  |
| **U6 gRNA cassette (synthetic gene cassette)** | | | |
|  | **ATTAAGGGTGAATGGCTACTAATTTAAATTTTAAGGATATTTTCTAGTAAATTCTATTTCATTTTGGTTCAAATGCAATATTTCTGAATCTTTTGAGCTTGTTGTTTCTTTTGACTTAGAGTCTATTGTGGAATGATGCTATATGAACCAAATCGATTTTGAAACATAAGAGATTTTGTCCAAAGAGTACCTTTGAATAATAAAATAGATTTCAAGGATTCTTTTAGGAAGGAGAAAGAGAATTTCTGCAATTTTCCATGATATAATCCATATATTTTTGTTTTTATTCTTTATATTCTATATGATATTTGAAGACAAAAACATTTTTAGTTTATAAAGCATTGTCAAACATATTCTATT*AGCTGAGGATATGGCCTAGT*GTTTTAGAGCTAGAAATAGCAAGTTAAAATAAGGCTAGTCCGTTATCAACTTGAAAAAGTGGCACCGAGTCGGTGCTTTTTTGACGTTCATTTCTGGATAGGTCCCATTTTTT** | | |
| **Note: Italicized = U6 seed sequence; Underlined = gRNA scaffold** | | | |
